# Enhancing implementation of measurement-based mental health care in primary care: a mixed-methods randomized effectiveness evaluation of implementation facilitation

**DOI:** 10.1186/s12913-018-3493-z

**Published:** 2018-10-03

**Authors:** Laura O. Wray, Mona J. Ritchie, David W. Oslin, Gregory P. Beehler

**Affiliations:** 1 Department of Veterans Affairs, VA Center for Integrated Healthcare, 3495 Bailey Avenue, Buffalo, NY 14215 USA; 2 Jacobs School of Medicine and Biomedical Sciences, University at Buffalo, 955 Main Street, Suite 6186, Buffalo, NY 14203 USA; 3 Department of Veterans Affairs, VA Quality Enhancement Research Initiative (QUERI) for Team-Based Behavioral Health, 2200 Ft Roots Dr, Bdg 58, North Little Rock, AR 72114 USA; 4 Department of Psychiatry, University of Arkansas for Medical Sciences, 4301 W Markham St, #755, Little Rock, AR 72205 USA; 5 VISN 4 Mental Illness Research, Education, and Clinical Center, Corporal Michael J. Crescenz VA Medical Center, 3900 Woodland Avenue, Philadelphia, PA 19104 USA; 6 Department of Psychiatry, Perlman School of Medicine, University of Pennsylvania, 3900 Chestnut St, Philadelphia, PA 19104 USA; 7 Schools of Public Health and Health Professions, University at Buffalo, 401 Kimball Tower, 955 Main Street, Buffalo, NY 14214 USA

**Keywords:** Implementation, Facilitation, Measurement-based care, Integrated primary care, Mental health, Primary care

## Abstract

**Background:**

Mental health care lags behind other forms of medical care in its reliance on subjective clinician assessment. Although routine use of standardized patient-reported outcome measures, measurement-based care (MBC), can improve patient outcomes and engagement, clinician efficiency, and, collaboration across care team members, full implementation of this complex practice change can be challenging. This study seeks to understand whether and how an intensive facilitation strategy can be effective in supporting the implementation of MBC. Implementation researchers partnering with US Department of Veterans Affairs (VA) leaders are conducting the study within the context of a national initiative to support MBC implementation throughout VA mental health services. This study will focus specifically on VA Primary Care-Mental Health Integration (PCMHI) programs.

**Methods:**

A mixed-methods, multiple case study design will include 12 PCMHI sites recruited from the 23 PCMHI programs that volunteered to participate in the VA national initiative. Guided by a study partnership panel, sites are clustered into similar groups using administrative metrics. Site pairs are recruited from within these groups. Within pairs, sites are randomized to the implementation facilitation strategy (external facilitation plus QI team) or standard VA national support. The implementation strategy provides an external facilitator and MBC experts who work with intervention sites to form a QI team, develop an implementation plan, and, identify and overcome barriers to implementation. The RE-AIM framework guides the evaluation of the implementation facilitation strategy which will utilize data from administrative, medical record, and primary qualitative and quantitative sources. Guided by the iPARIHS framework and using a mixed methods approach, we will also examine factors associated with implementation success. Finally, we will explore whether implementation of MBC increases primary care team communication and function related to the care of mental health conditions.

**Discussion:**

MBC has significant potential to improve mental health care but it represents a major change in practice. Understanding factors that can support MBC implementation is essential to attaining its potential benefits and spreading these benefits across the health care system.

## Background

Most modern medical care uses objective measurement to guide and evaluate treatment. For example, blood pressure measurement is routinely used to screen for hypertension, to determine if treatment is indicated, and to guide treatment including behavior-focused efforts. By contrast, modern mental health care typically uses subjective clinician assessment as the most common tool for guiding both psychotherapy and pharmacotherapy treatment decisions. Although reliable and valid patient reported outcome measures (PROM) for mental health conditions are available, their use to guide treatment beyond screening and initial evaluation is relatively infrequent [1–7]. Routine use of PROM to track patient symptoms, guide treatment decisions and facilitate communication between patients and providers is referred to as measurement-based care (MBC). There are three critical elements to MBC: (1) *collection* of information using psychometrically-sound self-report instruments repeatedly over the course of treatment; (2) use of that information to *guide treatment* decisions; and, (3) *sharing* the information with patients and others on the health care team to support collaborative treatment decision-making.

The evidence for MBC is strong when it is used over time in a systematic way to adjust treatment (pharmacotherapy or psychotherapy) rather than simply fed back to providers or patients (monitoring alone) [8–11]. In addition to its potential benefit to individual patient outcomes, MBC can improve treatment fidelity [9, 10, 12], improve patient-provider communication [13, 14], and increase patient engagement [15]. At the clinic and program level, MBC can support treatment team communication about mental health conditions [16, 17] and can facilitate quality improvement (QI) efforts [18] by providing patient outcome data that can be monitored in response to systematic changes in clinical operations. At the population level, MBC can improve the programmatic efficiency of care by identifying patients in need of more treatment and reducing the number of sessions for patients who have improved [9]. Further, MBC does not require new staffing and, while a MBC approach requires providers to alter their clinical practice, it does not add time to patient encounters [11]. As a result, while MBC improves the outcomes of care, it can also increase clinician efficiency [11]. Despite these benefits and calls for the implementation of standard systems of MBC in mental health practice, few health care systems have adopted it as a standard of care [11, 19, 20]. In fact, it is the norm for mental health providers in all clinic settings to rely heavily on clinical interviews focused on the individual experience of patients (ideographic assessment) rather than using standardized instruments that allow comparison to a normative group (nomothetic assessment) [5, 6, 21].

Beginning in 2015, the U.S. Department of Veterans Affairs (VA) began an initiative to increase the use of MBC throughout VA mental health services. The initiative has occurred in phases. In the first phase, leadership representing the broad scope of VA mental health care agreed on a set of standards that would define successful implementation. These standards include: the use of four specific PROMs (the Patient Health Questionnaire-9 (PHQ-9) [22], the Generalized Anxiety Disorder-7 (GAD-7) [23], the Brief Addiction Monitor (BAM) [24], and the Post Traumatic Stress Disorder Checklist-5 (PCL-5) [25, 26]); the requirement that assessment responses be accessible in the electronic health record and not just recorded in progress notes; and the collection of measures at the start and periodically throughout episodes of care. Having laid this groundwork, the next phase included a set of activities that have become standard in the VA system for supporting practice change initiatives. This national support included: developing educational materials for providers and patients, engaging volunteer programs throughout the system to begin implementation, supporting a community of practice for engaged sites, and, upon request, brief coaching for implementation problem-solving. That stage was recently completed with evidence of increased use of PROM throughout the healthcare system. Educational materials, web-based community of practice discussion, and expert consultation remain available. The next phase requires every facility to implement MBC in at least one clinical program. Coincident with these policy changes the Joint Commission is also now requiring the use of MBC in some behavioral health programs such as addiction and residential services [27].

The VA has been an early adopter of MBC in its Primary Care Mental Health Integration (PCMHI) program. Beginning in 2007, the program integrated mental health services into primary care clinics system-wide [28] using both care managers in a collaborative care model [29, 30] and embedded behavioral health providers (BHPs) in an integrated primary care model [31]. Collaborative care models of primary care treatment for depression provided early, evidence-based examples of how MBC can improve communication, collaboration, and quality of care [7, 29, 32]. While MBC is a core component of care management, embedded BHPs have typically relied on PROM only as part of an initial assessment; follow-up assessments are most often idiographic [5, 6, 21]. Further, the implementation of the BHP component of PCMHI has been more prevalent than the implementation of care management [33]. As a result, at many sites, the PCMHI program is not attaining the full benefit of MBC.

Even when PROM is used in PCMHI programs, it is often only partially used. For instance, measures may be collected using pen and paper but not transcribed into the electronic medical record. Thus, the data are unavailable for communication, team decision-making, and program improvement efforts. Further, in PCMHI, where providers are intended to be fully integrated members of primary care, this partial use of MBC limits the ability of the rest of the primary care team to coordinate with and support mental health treatments and presents a barrier to interdisciplinary team function [34]. Team communication is a crucial component of team development and is thought to be critical to establishing high performance health care delivery teams [20, 34, 35]. The implementation of MBC has the potential to provide a pathway to improved communication about mental health conditions, allowing professionals from medical and mental health disciplines to work in a more fully integrated manner [36, 37].

In summary, while MBC has significant potential to yield improved patient care and interdisciplinary practice, it is a complex practice that has proven to be challenging to fully implement. Therefore, the current protocol will seek to determine if external facilitation (EF) when combined with an internal QI team will improve the implementation of MBC practice as compared to sites receiving only standard national support. External facilitation, an evidence-based, multi-faceted process of interactive problem-solving and support [38, 39], can incorporate multiple other discrete implementation strategies, e.g., audit and feedback, education, and marketing [40–42], to address the challenges of implementing MBC for mental health conditions. Engaging stakeholders and involving them in implementation processes is a core component of facilitation [43, 44] and is critical for implementation success [45, 46]. QI teams in this study will be composed of local stakeholders who will share responsibility for implementation efforts, thus acting as internal facilitators. The Enhancing Implementation of MBC in primary care study therefore has two primary research aims:

- Aim 1: To understand whether and how an EF + QI team strategy can be effective in supporting the implementation of MBC as compared to standard VA national support.
- Aim 2: To explore the association of MBC practice with primary care and PCMHI communication and team functioning.

## Methods/design

This study employs a quasi-experimental multiple case study design [47], using mixed methods to accomplish the study aims. In addition to addressing the two primary aims, the study is designed to explore factors that may affect successful implementation.

### Study partners

This study was designed as a collaborative effort between VA national leaders and the study team with the shared goal of increasing the use of MBC in PCMHI programs. In order to realize this ongoing collaboration, a Partnership Panel meets regularly with study leaders to inform the project’s work, provide input into study procedures, e.g., site identification and matching, and, discuss emerging findings and their implications for both the project and system-wide implementation. The panel consists of members whose role in the VA includes relevant national leadership responsibilities and three PCMHI site leaders whose facilities have demonstrated high PROM utilization.

### Conceptual frameworks

**Fig. 1:**
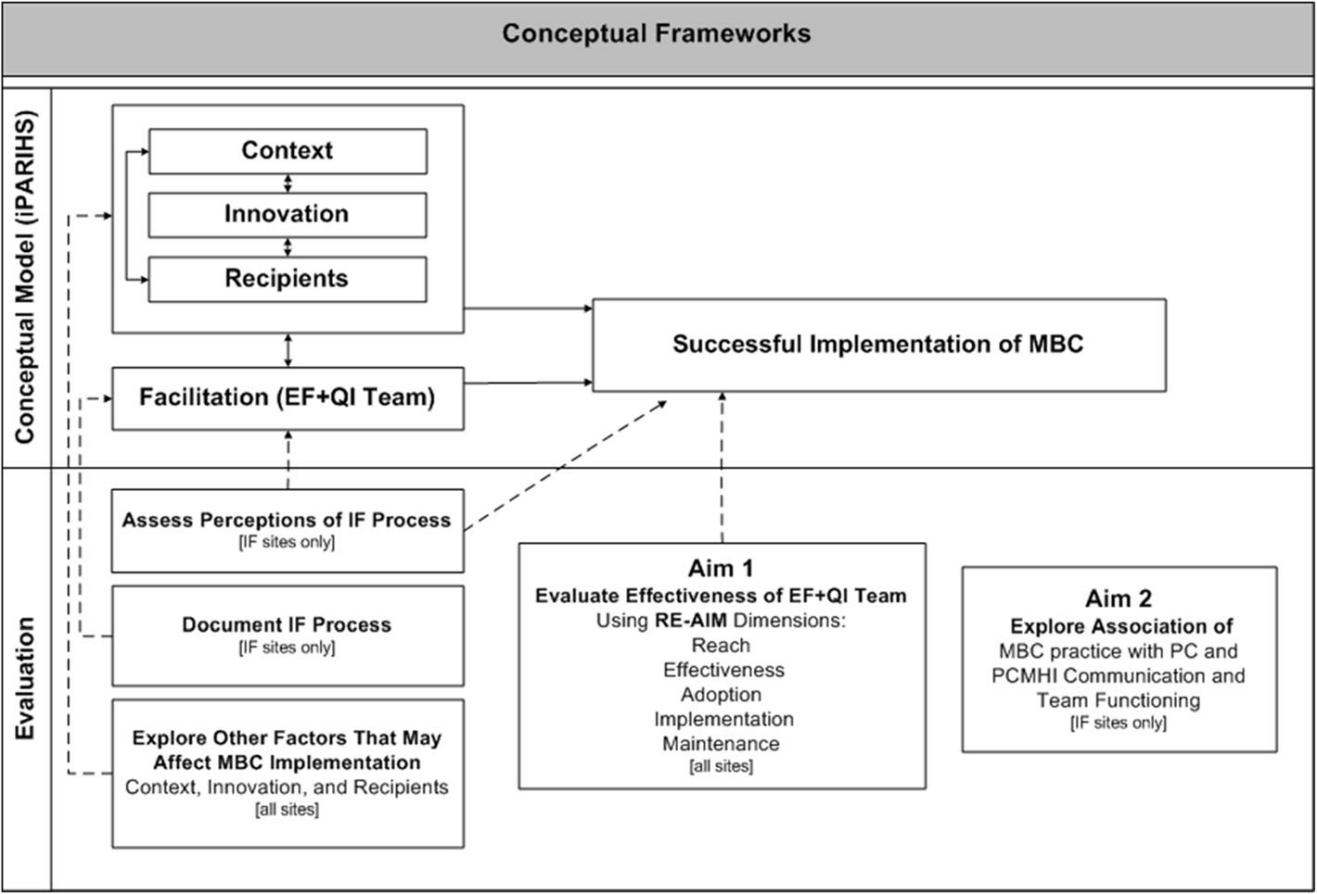
Conceptual frameworks guiding study design. RE-AIM guides evaluation of the IF strategy’s effectiveness and iPARIHS guides the design of the IF strategy and the evaluation of factors that may impact implementation. Legend: *EF* external facilitation, *IF* implementation facilitation, *MBC* measurement-based care, *QI* quality improvement

The RE-AIM [48] and integrated Promoting Action on Research Implementation in Health Services (iPARIHS) [49, 50] frameworks guided the design of this protocol. Figure 1 shows how these frameworks work together in the evaluation of the EF + QI team implementation strategy and factors that may affect implementation.

The RE-AIM framework guided the design of the evaluation of the implementation strategy’s effectiveness, i.e., the extent to which MBC is implemented at each site. RE-AIM is a useful framework for evaluating implementation interventions because it addresses issues related to real-world settings and assesses multiple dimensions (*Reach, Effectiveness, Adoption, Implementation, and Maintenance*). *Reach* is defined as the absolute number or proportion of eligible individuals who received the evidence-based practice. *Effectiveness* is defined as the impact of the evidence-based practice on identified outcomes. *Adoption* is defined as the absolute number or proportion of settings or staff using the evidence-based practice. *Implementation* is defined as the fidelity of the evidence-based practice or the proportion of its components as implemented in routine care. *Maintenance* is defined as the degree to which the evidence-based practice is sustained over time [48, 51]. Study measures will address each of the five RE-AIM dimensions to test the implementation strategy’s overall effect [52].

The iPARIHS framework guided the design of the implementation facilitation strategy and the assessment of factors that may influence MBC implementation. The iPARIHS framework suggests that four dimensions need to be considered when conducting implementation efforts [50]. Characteristics of three of these dimensions can hinder or foster implementation: *Innovation*, the focus of implementation efforts; *Recipients*, the individuals and teams who are affected by or who influence the implementation; and *Context*, factors within the local clinic, organization within which it is embedded, and the outer context, i.e., the wider health system. The fourth dimension, *Facilitation*, consists of a role, i.e., a designated person serving as a facilitator, and a process, the strategies and actions that a facilitator applies [40, 53]. *Facilitation* is the active ingredient that can maximize the potential for successful implementation by assessing and addressing challenges and leveraging strengths in each of the other dimensions.

### Site selection and recruitment

A rolling site recruitment approach is used so that four sites will begin study participation every 3 months. Site selection and recruitment processes for each cycle consists of three steps: identification of potential sites, stratification of similar sites into groups, and then recruitment of one or more pairs of sites within those groups. Initially, potential sites are drawn from a pool of 21 VA facilities that self-nominated their PCMHI program to participate in the first phase of the VA National MBC Initiative. In collaboration with the Partnership Panel, we selected administrative data variables (e.g., current use of PROM, site size, staffing and PCMHI program metrics [same-day access and population penetration]), and weighting current PROM use more heavily, we used these variables to stratify sites into relatively similar groups. Prior to each new recruitment cycle, we will draw the same administrative data, excluding participating study sites, to refresh the group placement and the Partnership Panel will review and provide input. If we are unable to recruit 12 sites from within the original pool, we will use the same administrative data variables and collaboration process to identify additional potential sites and group them by similarity. We will continue this process until all 12 sites have been selected and recruited into the study. For each cycle, we will recruit four sites whose PCMHI, primary care and mental health leaders have agreed to participate in study activities. Sites will be randomly assigned to either continued standard national support or the study implementation intervention.

### Participants

Within each of the 12 study sites, study participants will be predominantly VA employees selected based on their role in the clinics: PCMHI leaders, PCMHI providers, primary care providers and nurses serving on their teams, and QI team members. In addition to the PCMHI leader, QI teams will include the site’s primary care and mental health leaders or their designees, other members as identified by the site for inclusion, and Veteran patient representatives who may or may not be VA employees. Age, race/ethnicity, and gender distribution of VA staff will be reflective of those distributions among VA staff at participating clinics.

### Implementation facilitation strategy

The implementation facilitation strategy at the six intervention sites will combine expert external facilitation (EF) with an internal QI team to increase the use of MBC for mental health conditions within primary care. A team providing EF at each site will consist of an external facilitator and two to three MBC subject matter experts (SME). The two study external facilitators have expertise in implementation science principles and methods broadly, as well as PCMHI models of care. They each have extensive experience facilitating implementation of evidence-based innovations. Each will lead a facilitation team at intervention sites in supporting QI team members’ efforts to implement MBC.

The facilitation strategy will be applied at each site across three phases, Preparation Phase, Design Phase, and Implementation Phase.

#### Preparation phase

During this approximately 2-month long phase, the site facilitation team will engage leadership, begin to assess site needs and resources, and help identify QI team members.

#### Design phase

At the beginning of this approximately 3-month long phase, the external facilitator will conduct an in-person site visit during which he or she will meet with site leaders, the QI team and clinical staff to introduce the study, address gaps in MBC knowledge and perceptions of the evidence, and begin the process of planning for MBC implementation. Although the external facilitator will continue to engage local site leadership throughout this phase, the facilitation team will focus on 1) providing training to the QI team on PROM use, MBC practice and systems change, and 2) helping the QI team develop an implementation plan that is customized to local stakeholder needs, resources and preferences and then assess implementation status and needs. The implementation plan will include a kick-off date and process for the beginning of the next phase.

#### Implementation phase

At the beginning of this phase, site stakeholders, including the QI team, will meet to formally initiate the implementation of their plan. The external facilitator will be available through virtual technology to support the QI team in this kick-off meeting. Throughout this approximately 6-month phase, the QI team, with external facilitator and SME support, will educate, mentor, and encourage clinicians as they change their practice. They will also monitor implementation progress, conduct problem-identification and resolution, and help sites make adjustments to local MBC practices to overcome problems and enhance sustainability of MBC.

Across the intervention strategy phases, the facilitation team will gather information directly from site leaders and personnel and other sources (e.g., a national dashboard developed for the MBC initiative; local program level reports) for use in developing facilitation activities and to provide feedback to the QI team. Results of three baseline study measures, the Team Development Measure, the Organizational Readiness to Change instrument, and the Survey of MBC use, will also be provided to the facilitation team.

### Timing of data collection activities

**Fig. 2:**
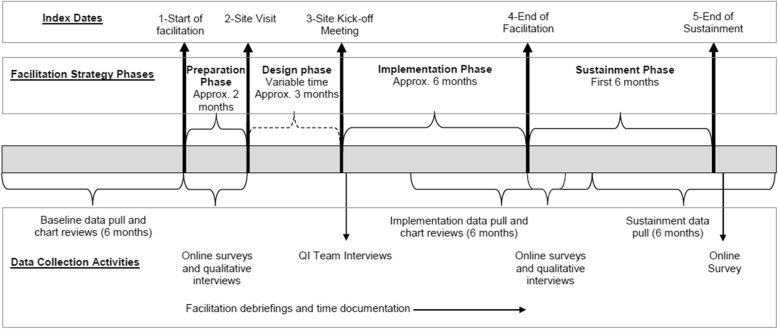
Study timeline for each site. Data collection activities queue off of index dates linked to facilitation events that demarcate the beginning or end of one of the phases of the implementation facilitation strategy

The timing of all data collection activities is based on index dates linked to facilitation activities that demarcate the phases of the facilitation strategy (Preparation Phase, Design Phase, and Implementation Phase) and an additional Sustainment phase, a 6-month observation period following the end of the facilitation intervention (see Fig. 2). Index dates at intervention sites will be used to structure evaluation activities for both the intervention sites and matched comparison sites.

Index Date 1 is defined as the date the external facilitator begins working with intervention site personnel. Index Date 2 is defined as the date of the external facilitator’s in-person site visit. Index Date 3 is defined as the date of the intervention site kick-off meeting. Index Date 4 is defined as the date that the external facilitator reports that the facilitation team has completed active facilitation and has ended regularly scheduled implementation support activities. Index Date 5 is defined as 6 months after the end of the Implementation Phase index date.

### Aim 1 data collection

Mixed methods will be used to 1) evaluate the effectiveness of the EF + QI team strategy to improve MBC implementation in primary care clinics and 2) assess factors that may affect MBC implementation.

#### Effectiveness of EF + QI team strategy

The RE-AIM framework guided the selection of evaluation measures to assess the effectiveness of the EF + QI team implementation strategy. For both intervention and comparison sites, data sources and measures were selected for each RE-AIM framework dimension (see Table 1). For intervention sites, additional data sources will provide supplementary information on the *Implementation* dimension. Data collection methods for the measures of RE-AIM dimensions follow.

**Table 1:** RE-AIM measures and data sources

RE-AIM Dimension	Measures	Data Sources
Reach	Proportion of patients with at least 3 PROMs documented during the first 6 months of care	Administrative data
Effectiveness	The impact of PROM use on treatment planning, team and patient communication	Chart reviews
		MBC qualitative interviews
		Provider surveys: Team Development Measure
		Provider surveys: MBC Use and Attitudes
Adoption	Proportion of staff who use PROM for care delivered	Administrative data
Implementation	The degree to which all 3 critical MBC elements ((1) collection, (2) use to guide treatment, and (3) sharing PROM with patients and providers) were implemented	Administrative data
		Provider surveys: MBC Use and Attitudes
		Chart reviews
		MBC qualitative interviews
		QI team interviews
		Debriefing interviews
Maintenance	Repeat measures 6 months later	Administrative data and Provider surveys: MBC Use and Attitudes

- **Administrative data**Administrative data focusing on the mental health services delivered in primary care to patients seen by PCMHI providers at all 12 sites will be pulled from the VA Corporate Data Warehouse to capture measures of *Reach*, *Adoption*, *Implementation*, and *Maintenance*. The study will access use of PROM and medical care utilization records for three time periods based on the site index dates: baseline, during the implementation, and during sustainment.

- **Chart reviews**To assess *Effectiveness and Implementation*, data will be extracted from randomly selected subsamples of patient records in the baseline and implementation administrative data pulls for all 12 sites (see Fig. 1). Electronic records of PROM administration and progress notes will be reviewed for each patient encounter during the observation period to extract data for the following variables: patient and provider demographics, dates of encounters, clinic locations, diagnostic codes, mental health PROM use and scores, and text pertaining to assessment and measurement administration/utilization. Progress notes will be reviewed for both the presence and absence of MBC elements, such as evidence that the PROM data played a role in clinical decisions and/or was communicated to the patient or other providers.

- **Provider survey: Measurement-based Care Use and Attitudes**Online surveys will be conducted with PCMHI and primary care providers and nurses to capture MBC use (*Effectiveness* and *Maintenance*) at all 12 sites at three time points (see Table 2 and Fig. 1). The survey instrument (Oslin DW, Hoff R, Mignogna J, Resnick SG: Provider attitudes and experience with measurement-based mental health care, submitted), developed for the VA National MBC Initiative and adapted for this study, will assess provider perceptions and attitudes about MBC and frequency of PROM use at key points in care.

**Table 2:** Data sources: Factors that may affect MBC implementation

	Facilitation	Context	Innovation	Recipients
Provider survey: MBC Use and Attitudes^a^		X	X	X
Provider survey: TDM^a^		X	X	X
MBC qualitative interviews^a^		X	X	X
Debriefing interviews (IF sites)^a^	X	X	X	X
Time data (IF sites)	X			
QI team interviews (IF sites)^a^	X	X	X	X
Provider survey: ORC		X		
Provider survey: PPAQ-2		X		
Team communication interviews		X		X

*ORC* Organizational Readiness to Change, *TDM* Team Development Measure, *PPAQ-2* Primary Care Behavioral Health Provider Adherence Questionnaire-2

^a^Data sources that also address RE-AIM dimensions evaluating the implementation strategy

- **Provider survey: Team Development Measure (TDM)**The TDM, which has strong psychometric properties, was designed to measure and promote quality improvement in team-based healthcare settings [54]. It will be administered online to providers at two time points (see Fig. 1 and Table 1). TDM data will be used to assess the degree to which health care teams at all sites have and use elements (cohesion, communication, role clarity and goals and means clarity) needed for highly effective teamwork (*Implementation*).

- **Measurement-based care qualitative interviews**We will conduct semi-structured qualitative interviews with PCMHI programmatic leaders at all 12 sites to assess the status of MBC implementation and use of MBC in current practice (*Effectiveness* and *Implementation*). These interviews will be conducted at two time points (see Table 2 and Fig. 1). Both interviews will focus on PCMHI leader perceptions of how PCMHI providers are collecting, using and sharing PROM, the evidence for MBC, the importance and value of MBC to site stakeholders, and the barriers and facilitators to MBC implementation. The second interview is designed to assess changes in these perceptions and attitudes, adaptations in clinic MBC practice, and additional barriers and facilitators to implementing MBC experienced since the last interview. Finally, we will ask for PCMHI leader views on potential barriers and facilitators to sustaining MBC if it has been implemented.

#### Factors that may affect MBC implementation

The iPARIHS framework guides our assessment of factors that may affect implementation. Table 2 shows data sources that will provide information for the assessment of the iPARIHS dimensions. Some of these data sources also address measures for the RE-AIM evaluation of the implementation facilitation (IF) strategy and were previously described.

##### Facilitation

Assessment of facilitation includes both self-reported documentation of activities by the external facilitators and SMEs, and, data collected from QI team members on IF activities. Because comparison sites will not receive facilitation, assessment of *Facilitation* will only be conducted at intervention sites.

- **Documentation of the facilitation strategy**Facilitation activities will be documented using two data collection mechanisms. First, we will conduct bi-weekly to monthly debriefing interviews with the external facilitators and SMEs and use summary notes to document on-going facilitation, QI team processes, and factors that might foster or hinder MBC implementation at each intervention site. Second, facilitation team members will document their time, activities, and numbers and roles of site personnel on activity logs in preparation for a time-motion analysis of facilitation activities. Debriefing interview and time-motion data will enable us to go beyond determining whether the EF + QI team strategy is effective and to offer possible explanations for why it is or is not effective within particular organizational contexts and how facilitators can enhance MBC implementation.

- **QI team interviews**To assess the facilitation process from the perspective of QI team members, semi-structured qualitative group interviews will be conducted with QI team members at intervention sites at two time points (see Fig. 2). The first interview will assess QI team accomplishments and functioning, the value of the facilitation process, and how it might be improved. The second interview will focus on MBC implementation and factors that affected it, results of and concerns about using MBC, MBC sustainment, and experiences with facilitation.

##### Context

We will assess select organizational factors at all 12 study sites with two validated instruments to enhance our understanding of contextual factors that may have affected MBC implementation.

- **Provider survey: Organizational Readiness for Change (ORC)**To assess baseline organizational functioning and climate at intervention and comparison sites, we will administer the ORC, which is considered to have good validity [55]. Similar to other VA implementation studies [56, 57], we will minimize participant burden by administering only ten of the ORC subscales across three domains, including: motivation to change subscales (program needs, training needs, and pressures for change), an adequacy of resources subscale (staffing), and, organizational climate subscales (mission, cohesion, autonomy, communication, stress, and, change) [58].

- **Provider survey: Primary Care Behavioral Health Provider Adherence Questionnaire-2 (PPAQ-2)**The PPAQ-2 is a scale of behavioral health provider protocol adherence to PCMHI. This adherence is thought to be essential to BHPs’ ability to function as part of the primary care team. The original PPAQ has been shown to have strong psychometric properties [59]. The recently revised and expanded version, PPAQ-2, has shown excellent reliability and validity through confirmatory factor analysis [60]. The study will use the following content domains: Practice and Session Management; Referral Management and Care Continuity; Consultation, Collaboration, and Interprofessional Communication; Patient Education, Self-Management Support, and Psychological Intervention; and Panel Management. Higher PPAQ-2 scores indicate high fidelity to PCMHI.

- **Other data sources**In addition to the ORC and PPAQ-2, data sources addressing RE-AIM measures will also inform our understanding of the context for MBC implementation. For example, the team communication interviews and TDM will provide us with additional information about the organizational culture and the MBC qualitative interviews will provide us with information about the contextual barriers to and enablers of change for MBC implementation. At the intervention sites, the debriefing interviews will be particularly rich sources of information about the organizational context across organizational levels.

##### Innovation and recipients

Some of our data sources will provide us with information about the characteristics of the innovation, e.g., MBC interviews and MBC use surveys (underlying knowledge sources, clarity and usability). They will also provide us with information about the recipients, e.g., Team communication interviews and TDM (collaboration and teamwork); QI team interviews (motivation to change and beliefs) and MBC interviews (values). Again, the debriefing interview data will provide us with a rich description of the recipients and the innovation at intervention sites.

### Aim 2 data collection

To study the association between team communication and functioning and MBC implementation, individual semi-structured qualitative interviews will be conducted with primary care (providers and nurses) and PCMHI providers at the facilitation intervention sites. These interviews will be conducted at two time points (see Fig. 1). Interview items reflect constructs related to team functioning in health care settings [61]. These items are tailored specifically for the topic of caring for primary care patients with mental health conditions: communication across primary care and PCMHI providers; impact of MBC implementation on provider communication; cohesiveness among team members; perceptions of professional role clarity; and perceptions of common team goals. Additionally, during the second interview, providers’ attitudes about the acceptability, feasibility and utility of using MBC will be assessed.

### Data analysis

The data analysis plan is designed to allow for triangulation across data sources in order to inform the two study aims. General analytic approaches are described first, followed by the planned strategies for addressing the two study aims.

#### Aim 1 data analysis

Administrative data will be used to compare rates of PROM administration and entry into the EMR across intervention and comparison sites. The analytic strategy will be to compare rates between the intervention and comparison sites at the end of the intervention and after a sustainment period while controlling for the baseline rate of use. Chart review data will be analyzed by calculating descriptive statistics for patient background/demographics, PCMHI service utilization, patient diagnoses, and, PCMHI provider type. Descriptive statistics will also be used to characterize PCMHI provider documentation of MBC elements including administration of measures of interest (i.e., PHQ-9, PCL, BAM, GAD-7, others) and indicators of how scores from each administration were used (e.g., to support a diagnosis, provide feedback to patient). Analysis of variance (ANOVA) will be used to assess for changes in frequency in chart review MBC elements by group (intervention v. comparison site). Provider survey data will be analyzed at the site level as follows. Analysis of the MBC provider surveys will examine differences in attitudes and PROM use by professional group (physicians, nurses, social workers, psychologists, etc.) as well as site. In addition, we will examine change in attitudes over time comparing intervention to comparison sites. As a non-standardized measure, we will be limited to examining frequencies and distributions of responses. Using the TDM, we will calculate ratings for overall team development and the four subfactors of cohesiveness, communication, role clarity, and goals and means. These ratings will be used to assess differences in team development both between and within sites over time. Mean overall clinic/team ORC scores will be calculated as will means on each subscale. Site ORC scores will be considered when interpreting qualitative findings regarding status of MBC implementation. PPAQ-2 data will be analyzed using descriptive statistics and a series of ANOVAs to compare overall site fidelity to the PCMHI model and variations of PPAQ-2 domains across intervention and comparison sites and over time. A descriptive analysis of time data will be conducted, calculating hours spent by the EF and SMEs in support of MBC implementation. We will examine variation in IF time across clinics, types of IF activities, and over time.

Analysis of MBC and QI Team qualitative interview transcripts and facilitation team debriefing notes will be conducted separately using a rapid analysis approach. Text will be coded utilizing qualitative data analysis software, extracted, and summarized for each site to answer target research questions. Site summary results for interviews conducted at two time points will be examined for changes over time.

The application of mixed methods in this design provides the opportunity to advance our understanding of these site changes. Using qualitative data sources, the analysis will yield a more complete description of *how* MBC is implemented in practice at each site. Further, individual site context factors will be incorporated by using ORC, TDM and PPAQ-2 results during qualitative analysis to help explain differences in site implementation status of MBC. For example, it would be expected that sites with low readiness to change (ORC) or low levels of team development (TDM) would be less likely to successfully implement any new practice, including MBC. Conversely, if the EF + QI Team strategy is successful in addressing team development challenges, a given site may experience greater levels of MBC implementation success. Similarly, sites with low PCMHI model fidelity may struggle to keep up with the demands of fast paced primary care clinics and may find the addition of MBC practice is too challenging. Finally, provider attitudes regarding MBC are known to limit uptake [2, 62, 63]. The distribution of clinician attitude and perception responses at sites will be considered when comparing site implementation status of MBC.

#### Aim 2 data analysis

Transcripts from Team Communication and Functioning interviews will be analyzed utilizing a different rapid analysis approach which will include a review of each transcript and extraction of data into a summary template in order to compile results for each domain/theme of the interview guide. As there will be multiple Team Communication and Functioning interviews per site, a site summary will be created from the case-by-case summaries. Site summary results for interviews conducted at two time points will also be examined for changes over time. In addition to examining the association of MBC practice with primary care and PCMHI communication and team functioning across varying contexts, individuals and teams, our final analysis will also examine whether there are patterns in responses related to the level of fidelity to PCMHI.

### Trial status

As described, initial potential sites were identified and grouped with Partnership Panel input. From within each of two of these groups, two sites have been recruited. Within these pairs, one site was randomized to the implementation strategy and facilitation activities began in May 2018.

## Discussion

The evidence for the systematic use, over time, of PROM for mental health conditions is strong but MBC is seldom the standard of care, even in VA PCMHI programs where collaborative care management services are designed with MBC as a core component. This study will evaluate an implementation strategy, consisting of external facilitation and an internal QI team, for improving utilization of measurement-based mental health care and explore the associations of MBC practice with team communication and functioning. The EF + QI team strategy combines MBC and implementation science expertise with the expertise and participation of local stakeholders to develop and execute an MBC implementation plan tailored to site needs and resources. Grounding the study in the RE-AIM and iPARIHS frameworks will allow us to document the strategy’s effectiveness, as well as how facilitation was utilized to address barriers [63–65] and leverage facilitators related to characteristics of MBC for mental health, site stakeholders, and the organizational context. This knowledge will be helpful to both our VA mental health operations partners and to mental health care systems outside VA seeking to modernize mental health care delivery. Further, as MBC becomes a new standard of care [27], it will be essential to understand how this practice can be efficiently implemented and sustained.

This project is one of three linked studies focusing on team-based mental health care; the other two studies examine (1) the addition of veteran peer specialists to the primary care team [57] and (2) the implementation of interdisciplinary behavioral health teams in general mental health clinics [66]. This portfolio of projects, part of a VA Quality Enhancement Research Initiative (QUERI) funded program, QUERI for Team-Based Behavioral Health (BH QUERI; QUE 15-289), is responsive to the Institute of Medicine recommendation that “all health professionals should be educated to deliver patient-centered care as members of an interdisciplinary team, emphasizing evidence-based practice, quality improvement approaches, and informatics” [20]. Interdisciplinary team-based care, while valuable, is complex and challenging to implement [67]. Therefore, this group of projects was designed to study the application of implementation facilitation to address the challenges of implementing these innovations and complex care delivery models, including team-based care [41, 68, 69]. If successful, these projects may individually and collectively advance the understanding of key factors necessary to achieve the Institute of Medicine recommendations on team care. Additionally, in sites where MBC is implemented, this project has potential to advance our understanding of interprofessional team practice as we explore whether the development of a shared system of communication regarding mental health strengthens team-based primary care in integrated care settings.

As is often the case with implementation science studies, the evolving organizational context has been, and likely will continue to be, a challenge to this project. For example, between the time that this project was initially proposed and funding started, the VA national initiative efforts moved from planning to active stages resulting in the need to redesign components of the project. As a result, the project’s site recruitment plan was refocused on PCMHI sites that engaged in the VA National MBC Initiative and our comparison condition became standard national support. Our project was strengthened by these changes as they allow us to compare our resource intensive implementation support intervention to a ‘light touch’ standard support condition. If successful, our project will help us to answer an important question for our operations partners. That is, “How much support is necessary and sufficient to implement a complex new health care practice?” It is only through our close and ongoing partnership with the VA leaders who are responsible for national MBC implementation efforts that we were able to navigate the changing national context. Through continued close collaborations we hope to execute this study in a way that will result in meaningful findings.

## Abbreviations

ANOVA: Analysis of Variance
BAM: Brief Addiction Monitor
BH: Behavioral Health
BHP: Behavioral Health Provider
EF: External Facilitation
EMR: Electronic Medical Record
GAD-7: Generalized Anxiety Disorder-7
IF: Implementation Facilitation
iPARIHS: integrated Promoting Action on Research Implementation in Health Services
MBC: Measurement-based Care
ORC: Organizational Readiness for Change
PCL-5: Post-Traumatic Stress Disorder Checklist-5
PCMHI: Primary Care-Mental Health Integration
PHQ-9: Patient Health Questionnaire-9
PROM: Patient Reported Outcome Measure
QI: Quality Improvement
QUERI: Quality Enhancement Research Initiative
RE-AIM: Reach, Effectiveness, Adoption, Implementation and Maintenance
SME: Subject Matter Experts
TDM: Team Development Measure
VA: U.S. Department of Veterans Affairs
VISTA: Veterans Health Information Systems and Technology Architecture
